# PAS kinase deficiency reduces aging effects in mice

**DOI:** 10.18632/aging.102745

**Published:** 2020-01-23

**Authors:** Pilar Dongil, Ana Pérez-García, Verónica Hurtado-Carneiro, Carmen Herrero-de-Dios, Elvira Álvarez, Carmen Sanz

**Affiliations:** 1 Department of Biochemistry and Molecular Biology, Faculty of Medicine, Complutense University of Madrid, Institute of Medical Research at the Hospital Clínico San Carlos (IdISSC), Ciudad Universitaria, Madrid, Spain; 2 Spanish Biomedical Research Centre in Diabetes and Associated Metabolic Disorders (CIBERDEM), Madrid, Spain; 3 Department of Cell Biology, Faculty of Medicine, Complutense University of Madrid, Madrid, Spain

**Keywords:** oxidative stress, mitochondrial function, antioxidant enzymes, liver regeneration, hepatic ROS

## Abstract

Several signaling pathways may be affected during aging. All are regulated by nutrient levels leading to a decline in mitochondrial function and autophagy and to an increase in oxidative stress. PAS Domain Kinase (PASK) is a nutrient and bioenergetic sensor. We have previously found that PASK plays a role in the control of hepatic metabolic balance and mitochondrial homeostasis. To investigate PASK’s role in hepatic oxidative stress during aging, we analyzed the mitochondrial function, glucose tolerance, insulin resistance, and lipid-related parameters in aged PASK-deficient mice. Hepatic *Pask* mRNA decreased in step with aging, being undetectable in aged wild-type (WT) mice. Aged PASK-deficient mice recorded lower levels of ROS/RNS compared to aged WT. The regulators of mitochondrial biogenesis, PGC1a, SIRT1 and NRF2, decreased in aged WT, while aged PASK-deficient mice recorded a higher expression of NRF2, GCLm and HO1 proteins and CS activity under fasted conditions. Additionally, aged PASK-deficient mice recorded an overexpression of the longevity gene *FoxO3a*, and maintained elevated PCNA protein, suggesting that hepatic cell repair mechanisms might be functional. PASK-deficient mice have better insulin sensitivity and no glucose intolerance, as confirmed by a normal HOMA-IR index. PASK may be a good target for reducing damage during aging.

## INTRODUCTION

Aging can be defined as a multifactorial process characterized by a progressive decrease in physiological functions resulting from the lifelong accumulation of damage. Such damage could be mediated by, among others, oxidative stress, an impaired mitochondrial function, the deregulation of autophagy, and the shortening of telomeres [1].

A disturbance in the mitochondrial function and an increase in mitochondrial mass occur during aging [2]. This involves a progressive accumulation of damaged mitochondria and an altered cell function, as is also the case in other human pathologies [3].

As a consequence of mitochondrial function, reactive oxygen species (ROS) are actively produced. ROS, such as radical superoxide, non-radical hydrogen peroxide, and hydroxyl radicals, are highly reactive molecules that play contrasting roles. They can act as signal transducers not only in cell proliferation [4], but also in cellular senescence [5] or cell death [6]. Their excess causes oxidative stress, damaging essential cellular components such as DNA, proteins, and lipids [7, 8].

However, oxidative stress can be avoided by a cellular mechanism responsible for removing ROS, referred to as the antioxidant defense. It includes various antioxidant enzymes (i.e., superoxide dismutase (SOD), catalase (CAT), and glutathione peroxidase (GPx)), converting free radicals into oxygen and water. Besides antioxidant enzymes, there are also non-enzymatic molecules such as reduced glutathione (GSH) [7].

The balance between ROS and protective antioxidant responses is therefore an important factor in determining aging and lifespan, although the results are contradictory. SOD1 deficiency decreases life expectancy [9], while an overexpression of catalase increases the lifespan in mice [10]. Other reports, by contrast, have shown that an overexpression of antioxidant enzymes in mice, such as SOD1 or catalase, does not affect lifespan [11, 12].

The liver integrates metabolic machinery to meet energy demand during normal physiology as glycolysis and mitochondrial oxidative phosphorylation. This condition makes it more susceptible to oxidative stress damage. However, the liver also has powerful antioxidant mechanisms. A common characteristic of many chronic liver diseases nearly always involves increased oxidative stress, regardless of the cause of the liver disorder [13].

Several reports indicate that caloric restriction and intermittent periods of fasting may reduce the risk of complications associated with aging [14, 15]. Cells use nutrient sensing to identify and respond to differences in nutrient levels; the sensing mechanisms are dysregulated during the aging process [16]. AMP-activated protein kinase (AMPK) and the mammalian target of rapamycin (mTOR) pathways are nutrient sensors that have been involved in lifespan [17]. Additionally, PASK (a serine/threonine kinase that contains PAS domains) can sense intracellular oxygen, redox state, and various metabolites [18]. We have previously described how PASK is a critical regulator of AMPK and mTOR pathways in the hypothalamus, neuroblastoma N2A cells and liver [19, 20], as well as a key regulator of oxidative stress and glucose and lipid liver metabolism [21, 22]. PASK-deficient mice are protected against the development of obesity and insulin resistance induced by a high-fat diet (HFD) [23–25]. PASK has recently been described as a target of mTORC1 during regenerative myogenesis in muscle stem cells [26].

This report investigates the role of PASK in hepatic oxidative stress and the mitochondrial function under non-fasted and fasted conditions in step with aging, also analyzing the effect of PASK deficiency on glucose tolerance, insulin resistance, and lipid-related parameters in aged mice.

## RESULTS

### Aging effects on hepatic *Pask* gene expression

**Figure 1:**
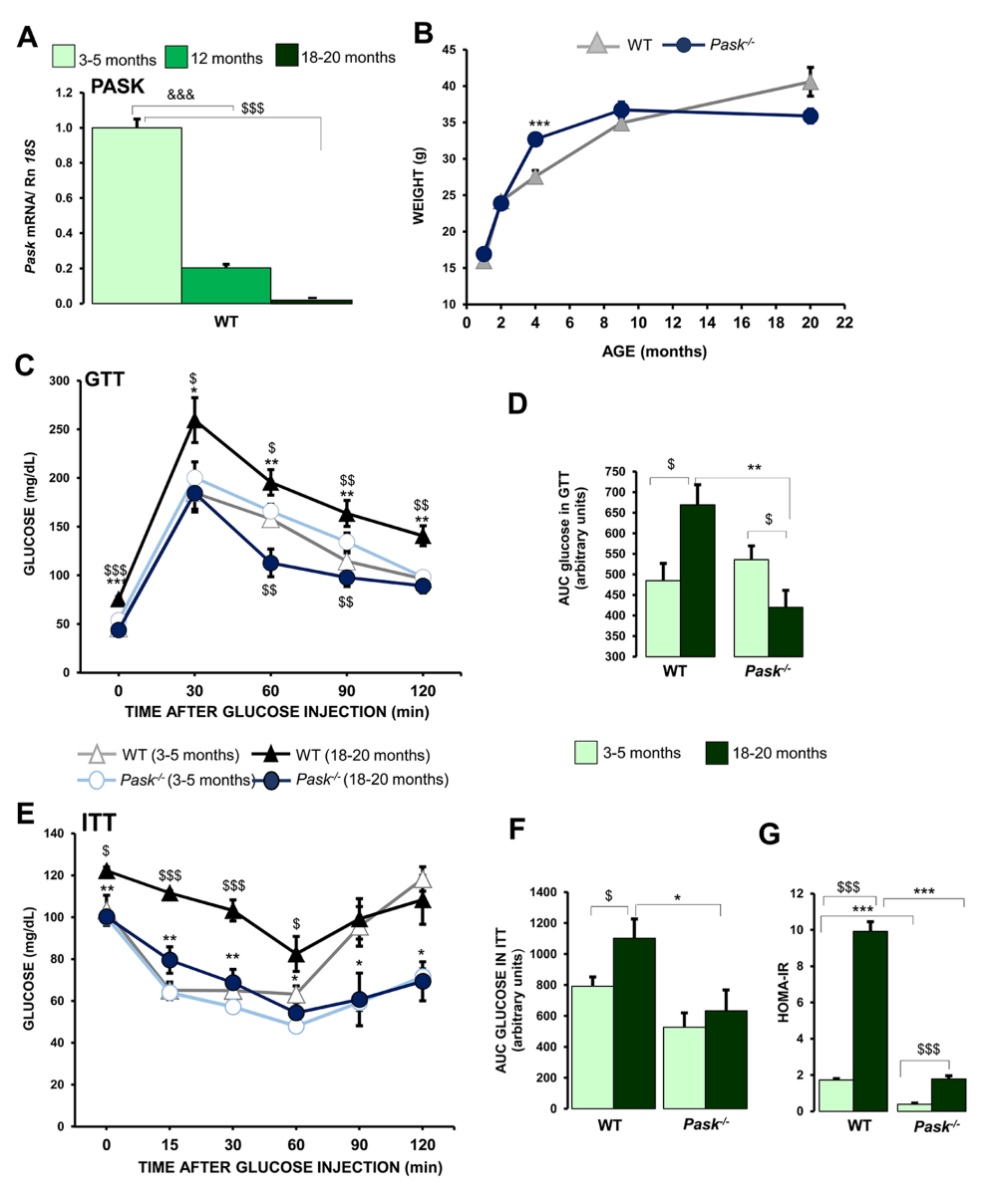
**Effects of aging in the regulation of hepatic PASK expression, and parameters affected by aging: glucose tolerance, action of insulin and lipid-related parameters.** Real-time PCR was used to analyze the expression of *Pask* (**A**) mRNA levels in livers from 3-5 months (young), 12-months, and 18-20 months (aged) wild-type (WT) mice. The value obtained for 3-5-month-old WT mice was taken as 1. ^&&&^ *P* < 0.001 3-5 months *vs*. 12 months; ^$$$^ *P* < 0.001 3-5 months *vs.* 18-20 months. (**B**) Comparison of growth curves of Δ WT and ● PASK-deficient mice, weight is means ± SEM. *** *P* < 0.001 WT vs. *Pask^-/-^* (**C**–**F**) Glucose and insulin tolerance tests (GTT/ITT); serum glucose levels (mg/dL) were measured before and several times after an IP glucose (**C**, **D**) or insulin (**E**, **F**) injection in mice of Δ 3-5 months or ▲18-20 months WT and ○ 3-5 months or ● 18-20 months PASK-deficient mice (*Pask*^-/-^). Line graphs represent the means ± SEM; n = 8-11 animals per condition. ^*$*^ *P* < 0.05, ^$$^ *P* < 0.01, ^$$$^ *P* < 0.001 3-5 months *vs*. 18-20 months; * *P* < 0.05, ** *P* < 0.01, *** *P* < 0.001 aged WT vs. aged *Pask^-/-^*. Area under the curve for glucose (AUC_glucose_) (**D**) and insulin (AUC_insulin_) (**F**). HOMA-IR values (**G**). (**D**, **F**, **G**) values are expressed as the means ± SEM in WT mice aged 3-5 months or 18-20 months and PASK-deficient mice (*Pask*^-/-^) aged 3-5 months or 18-20 months. ^*$*^ *P* < 0.05, ^$$$^ *P* < 0.001 3-5 months *vs*. 18-20 months; * *P* < 0.05, ** *P* < 0.01*,* *** *P* < 0.001 WT vs. *Pask^-/-^*.

The mRNA levels coding to PASK were measured by real-time PCR to analyze the effects of aging on *Pask* expression. We used livers from WT mice aged 3-5 months (young), 12 months, and 18-20 months (aged). The expression of mRNA coding to PASK decreased 80 % in 12-months-old mice, and was undetectable in aged WT mice (Figure 1A).

### Aging effects on growth curves in PASK-deficient mice

The growth curves revealed a greater weight gain between two to approximately eight months in PASK-deficient mice than in WT. Subsequently, ten-months-old PASK-deficient mice recorded a slow body weight loss, while WT mice maintained a slow body weight gain (Figure 1B).

### PASK deficiency blocks the development of glucose intolerance, insulin resistance, and alters lipid profile in aging

Aging is characterized by different changes at the physiological and molecular level over time, such as the development of a resistance to the action of insulin, changes in body composition, and alterations in the lipid profile, all leading to the development of type 2 diabetes.

Aged WT mice recorded high glucose levels under fasted conditions (75.6 ± 2.3 mg/dL) compared to young WT mice (45.6 ± 1.8 mg/dL). Nevertheless, aged PASK-deficient mice maintained similar glucose levels (43.9 ± 3.8 mg/dL) to young PASK-deficient mice (53.9 ± 4.8 mg/dL) (Figure 1C). The response of aged PASK-deficient mice to a glucose tolerance test (GTT) differed from that of the aged WT, which revealed an apparent glucose intolerance, with blood glucose levels being higher and remaining so for two hours after the glucose injection. However, the glucose levels in aged PASK-deficient mice were similar to or lower than both young WT and PASK-deficient mice (Figure 1C).

These results were confirmed by the areas under the curve calculated from TTG (AUC_glucose_). The AUC measured for aged WT was significantly higher than that measured for young WT. In contrast, the values from PASK-deficient mice were significantly lower in aged mice than in young and aged WT specimens. No significant difference was found between both groups of young mice (Figure 1D).

A significant increase in insulin levels was also observed in aged WT mice compared to young WT specimens, while insulin levels in aged PASK-deficient mice increased significantly less (Table 1). Likewise, improved insulin-sensitivity was confirmed by the insulin tolerance test (ITT) on aged PASK-deficient mice, being similar to both young PASK-deficient and WT mice (Figure 1E). The areas under the curves calculated from ITT (AUC_insulin_) increased significantly in aged WT compared to young mice. However, they were significantly lower in aged PASK-deficient mice than in aged WT mice, and similar to young mice (Figure 1F).

**Table 1:** Effects of aging and PASK deficiency on the blood insulin concentration, triglyceride and total cholesterol levels.

**Age (months)**	**mice**	**Insulin (ng/mL)**	**TCH (mg/dL)**	**TG (mg/dL)**
3-5	WT	0.84 ± 0.11	95.25 ± 7.03	119.60 ± 10.80
	*Pask^-/-^*	1.13 ± 0.12	97.50 ± 5.20	115.50 ± 12.60
18-20	WT	3.91 ± 0.12^$$$^	92.33 ± 4.00	125.70 ± 13.70
	*Pask^-/-^*	1.81 ± 0.05^$***^	70.57 ± 4.40^$$**^	82.71 ± 4.38^$**^

C57BL/6J (WT) and PASK-deficient (*Pask^-/-^*) mice. ^**^ *P* < 0.01; ^***^ *P* < 0.001 WT *vs*. *Pask^-/-^*; ^$^ *P* < 0.05; ^$$^ *P* < 0.01; ^$$$^ *P* < 0.001 3-5 months *vs.* 18-20 months. n=5-6.

Likewise, insulin resistance (as determined by HOMA-IR) was more than fivefold higher in aged WT compared to aged PASK-deficient mice (Figure 1G). Taken together, our results suggest that PASK-deficient mice were protected against developing age-dependent insulin resistance.

Lipid profile data showed that circulating triglyceride (TG) and total cholesterol levels (TCH) were similar in both aged and young WT mice. However, these levels significantly decreased in aged PASK-deficient mice (Table 1).

### Aging affects Akt activity differently in PASK-deficient mice

It has been reported that Akt plays a key role in the aging process through the regulation of energy metabolism. Insulin signaling begins with the autophosphorylation of the tyrosine residues of the insulin receptor generating docking sites for signaling proteins. Metabolic signaling is mediated through the PI3K/Akt pathway with the activation of phosphatidylinositol-3,4,5-triphosphate kinase (PI3K). Phosphoinositide-dependent kinase-1 (PDK1) activation induces the partial activation of Akt, and full activation requires Ser473 phosphorylation by other kinases (probably mTORC2), finally regulating multiple substrates. This pathway’s signaling effect depends also on the phosphatase PTEN. We analyzed the aging effect on the expression levels and activation of Akt and PTEN in liver from non-fasted and 24-h fasted WT and PASK-deficient mice.

**Figure 2:**
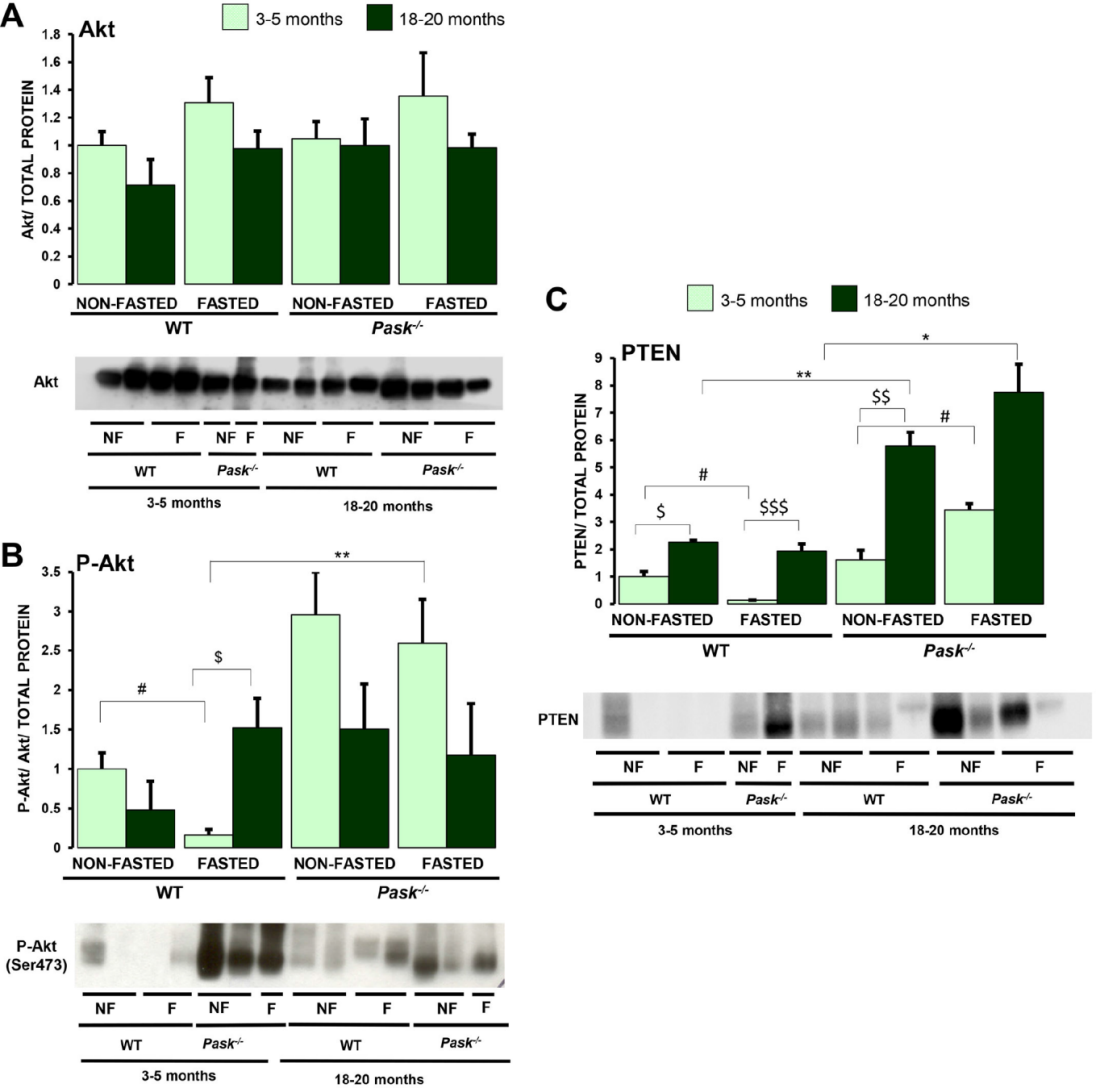
**Effects of aging and PASK deficiency on Akt and PTEN protein levels.** Immunoblot analysis of total Akt (Akt) (**A**), phospho-Akt (Ser473) (P-Akt) (**B**) and PTEN (PTEN) (**C**) in livers from young (3-5 months) and aged (18-20 months) wild-type (WT) and PASK-deficient (*Pask^-/-^*) mice. Liver lysates from non-fasted (NF) and 24-h fasted (F) mice were processed for western blot analysis. The value obtained for non-fasted 3-5-month-old WT mice was taken as 1. Bar graphs represent the means ± SEM of the densitometric values normalized by total protein detected by Stain-Free (TOTAL PROTEIN) (Supplementary Figure 1); n = 4-5 animals per condition. ^$^ *P* < 0.05, ^$$^ *P* < 0.01, ^$$$^ *P* < 0.001 3-5 months *vs*. 18-20 months; * *P* < 0.05, ** *P* < 0.01 WT *vs*. *Pask^-/-^*; ^#^ *P* < 0.05 non-fasted *vs*. fasted.

Our results indicate that aging slightly decreases the Akt activity in both WT and PASK-deficient mice. Nevertheless, while fasting severely inhibited Akt activity in young WT mice, fasted aged mice maintained a higher activity (Figure 2A, 2B). In contrast, PASK deficiency maintained a higher Akt activation under fasted conditions in young mice, and a similar albeit slight lower activation was observed in aged mice (Figure 2A, 2B).

Aging tended to upregulate the levels of protein PTEN, both in WT and PASK-deficient mice. Interestingly fasted aged WT mice maintained high PTEN levels in contrast to young mice with downregulated PTEN levels compared to non-fasted mice. Although a similar expression was found under basal conditions in both young PASK-deficient and WT mice, fasting increased PTEN protein levels in young PASK-deficient mice, while aging upregulated PTEN levels under basal conditions, and similar levels were detected in fasted aged PASK-deficient mice (Figure 2C).

### PASK deficiency alters the aging-dependent expression of certain genes involved in mitochondrial biogenesis

The aging process seriously affects the maintenance of the mitochondrial function and energy homeostasis. We therefore analyzed the expression of PGC1a and SIRT1 and the transcription factors regulated by PGC1a (NRF2, PPARg) that coordinate mitochondrial biogenesis and the expression of components involved in ATP production.

**Figure 3:**
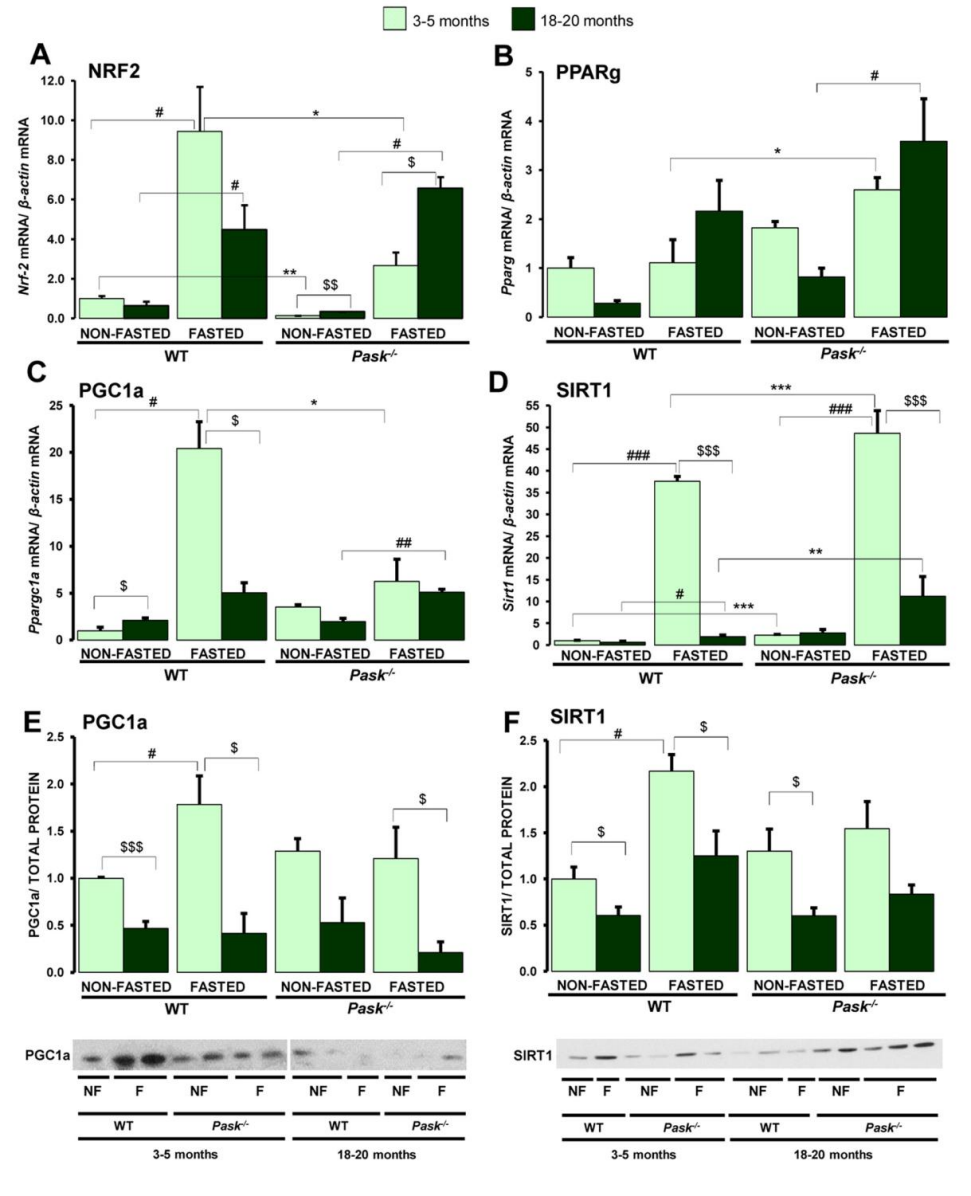
**Effects of aging and PASK deficiency on the expression of several hepatic genes involved in mitochondrial biogenesis and PGC1a and SIRT1 proteins.** Real-time PCR was used to analyze the expression of *Nrf2* (**A**), *Pparg* (**B**), *Ppargc1a* (**C**) and *Sirt1* (**D**) genes. The mRNA levels were measured in the livers of non-fasted (NON-FASTED) and 24-h fasted (FASTED) young (3-5 months) and aged (18-20 months) wild-type (WT) and PASK-deficient (*Pask^-/-^*) mice. Immunoblot analysis of PGC1a (**E**) and SIRT1 (**F**) in livers from young and aged WT and *Pask^-/-^* mice. Liver lysates from non-fasted (NF) and 24-h fasted (F) mice were processed for western blot analysis. The value obtained in 3-5-month-old non-fasted WT mice was taken as 1. Bar graphs in (**A**–**D**) represent the means ± SEM, and the levels of expression were normalized by mRNA of *β-actin* used as housekeeping gene;(**E**, **F**) represent the means ± SEM of the densitometric values normalized by total protein detected by Stain-Free (TOTAL PROTEIN) (Supplementary Figure 1); n = 4-5 animals per condition. ^$^ *P* < 0.05, ^$$^ *P* < 0.01, ^$$$^ *P* < 0.001 3-5 months *vs.* 18-20 months; * *P* < 0.05, ** *P* < 0.01, *** *P* < 0.001 WT *vs*. *Pask^-/-^*; ^#^ *P* < 0.05, ^##^ *P* < 0.01, ^###^ *P* < 0.001 non-fasted *vs.* fasted.

The expressions of *Nrf2, Ppargc1a* and *Sirt1* were upregulated by fasting in young WT mice. However, aged mice were unable to induce the expression of these genes up to the levels reached in young specimens, or the induction was mild in fasting (Figure 3A, 3C, 3D). Nevertheless, the expression of these genes was higher than in the non-fasted mice. The same effect was observed in PASK-deficient mice, although the *Sirt1* expression during fasting was higher than in WT mice. In the case of *Nrf2,* the expression was reinforced in both conditions of non-fasted and fasted aged PASK-deficient mice compared to young specimens, while the fasting effect was slightly better than in aged WT mice. In the case of *Pparg* (Figure 3B), aged mice showed a slight decrease in the expression under non-fasting conditions compared to young WT and PASK-deficient mice. In contrast, under fasting conditions, older mice showed a tendency to increase *Pparg* expression compared to young mice, which was only statistically significant in the case of PASK-deficient mice.

Regarding protein levels, aging decreased the expression of PGC1a and SIRT1 proteins in both WT and PASK-deficient mice, and no stimulation by fasting was observed in aged WT or PASK-deficient mice, in contrast to the effect observed in young WT mice (Figure 3E, 3F).

### The mitochondrial function modified by aging is PASK dependent

In order to evaluate the aging effect on mitochondrial biogenesis activity in liver from PASK-deficient mice, we analyzed some of the components of the machinery involved in different mitochondrial functions. PGC1a and other transcription factors and nuclear receptors regulate the expression of mitochondrial proteins, with an example being citrate synthase (CS). These mitochondrial enzymes have been tested to determine mitochondrial content and function, as well as certain genes in the electronic transport complex, such as cytochrome c oxidase subunit IV (COX-IV*)* and hepatic mitochondrial DNA content through the ratio of mitochondrial/nuclear DNA. Likewise, aging is characterized by an increase in oxidative stress, changes in the levels of expression of antioxidant enzymes, and mitochondrial dysfunction, seriously affecting cellular redox homeostasis. We therefore studied levels of hepatic ROS/RNS among young and aged WT and PASK-deficient mice.

**Figure 4:**
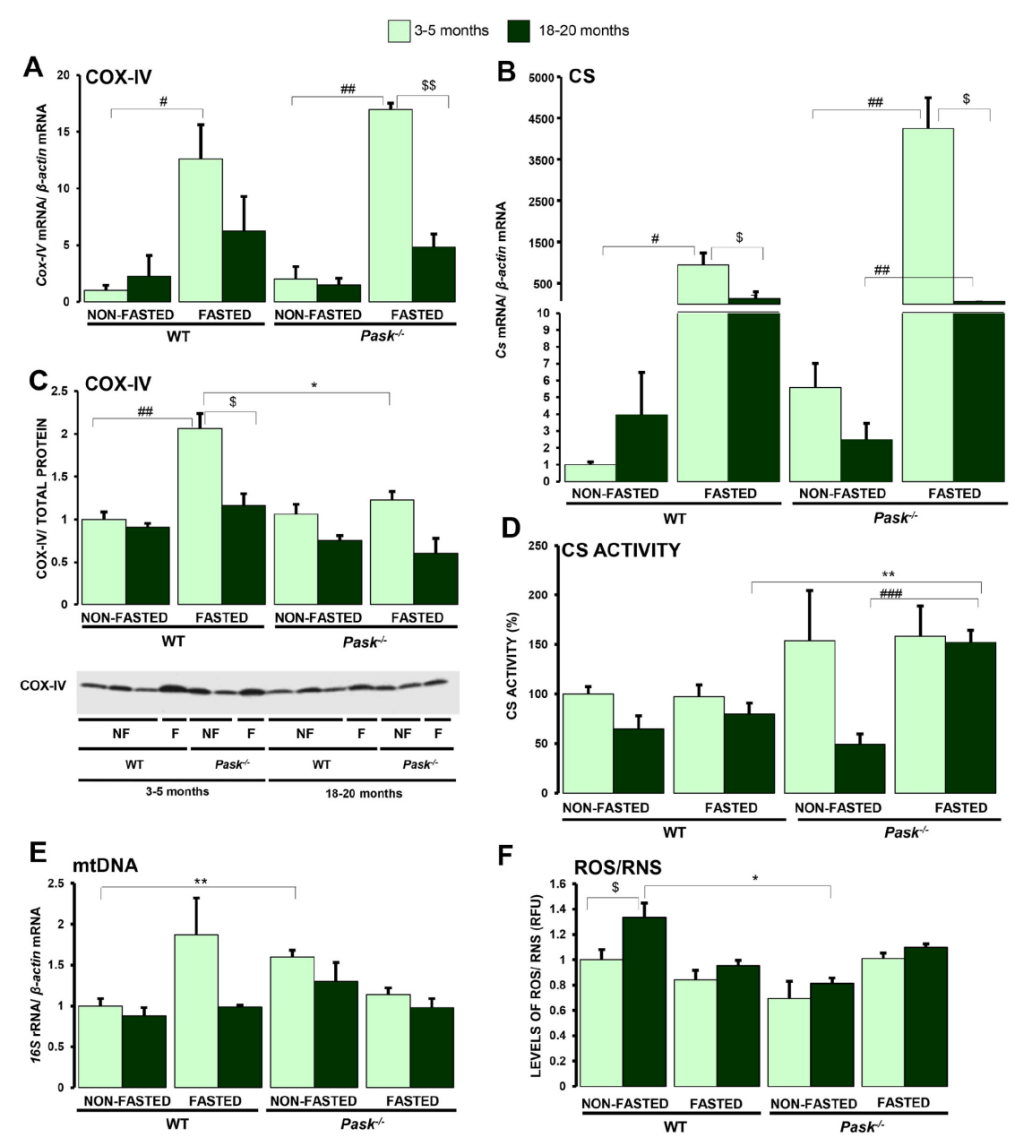
**Effects of aging and PASK deficiency on the expression and activity of certain mitochondrial proteins and ROS/RNS content in liver.** Real-time PCR was used to analyze the expression of *Cox-IV* (**A**) and *C*s (**B**) mRNA levels. The results were measured in the livers from non-fasted (NON-FASTED) and 24-h fasted (FASTED) young (3-5 months) and aged (18-20 months) wild-type (WT) and PASK-deficient (*Pask^-/-^*) mice. Immunoblot analysis of COX-IV (**C**) in livers from young and aged WT and *Pask^-/-^* mice. Liver lysates from non-fasted (NF) and 24-h fasted (F) mice were processed for western blot analysis. Citrate synthase activity (**D**) in liver homogenates was also measured. Mitochondrial DNA content (**E**) and the levels of ROS/RNS (**F**) were also measured. The value obtained in 3-5-month-old non-fasted WT mice was taken as 1. Bar graphs in (**A, B**) represent the means ± SEM, and the levels of expression were normalized by the mRNA of β*-actin* used as housekeeping gene; (**C**) represents the means ± SEM, of the densitometric values normalized by total protein detected by Stain-Free (TOTAL PROTEIN) (Supplementary Figure 1); (**D**) represents the means ± SEM, of citrate synthase activity, expressing them as a percentage; n = 4-5 animals per condition. Bar graphs in (**E, F**) represent the means ± SEM. ^$^ *P* < 0.05, ^$$^ *P* < 0.01 3-5 months *vs*. 18-20 months; * *P* < 0.05, ** *P* < 0.01 WT *vs*. *Pask^-/-^*; ^#^ *P* < 0.05, ^##^ *P* < 0.01, ^###^ *P* < 0.001 non-fasted *vs*. fasted.

The stimulatory effect of fasting on *Cox-IV* and *Cs* expression was lower in liver from aged WT and PASK-deficient mice compared to both groups of young mice, although *Cs* expression still increased significantly in fasted aged mice (Figure 4A, 4B). COX-IV protein levels were similar under basal conditions in young and aged WT, and similar levels were found in fasted aged WT mice, albeit lower than in young WT. However, similar protein levels were observed under both basal and fasted conditions in both young and aged PASK-deficient mice, even though a slight but not significant decrease was detected in aged PASK-deficient mice (Figure 4C).

Citrate synthase activity in liver homogenates tended to decrease with aging under basal conditions in both aged WT and PASK-deficient mice. However, although no significant differences were detected in CS activity under 24-h fasted conditions in aged WT, aged PASK-deficient mice still recorded higher levels of activity for this enzyme (Figure 4D).

Mitochondrial DNA content was similar in both aged WT and PASK-deficient mice compared to young mice, and no significant effects of fasting were observed in either case (Figure 4E).

However, lower ROS/RNS levels were found in aged PASK-deficient mice compared to aged WT specimens, while under basal and fasted conditions ROS/RNS content was similar in young and aged PASK-deficient mice. ROS/RNS levels, however, slightly increased in aged mice compared to young WT (Figure 4F).

### PASK deficiency reduces the inhibition of age-related antioxidant gene expression.

Controlling oxidative stress also depends on the expression of antioxidant enzymes. We analyzed the effect of PASK deficiency on the mRNA levels of ROS detoxification machinery: Catalase (CAT), Superoxide Dismutase (SOD: mainly mitochondrial MnSOD and Cu/ZnSOD located in the cytosol) and Glutathione Peroxidase (GPx). SOD transforms superoxide radicals into either ordinary molecular oxygen (O_2_) or hydrogen peroxide (H_2_O_2_), and CAT degrades H_2_O_2_, being mainly located in peroxisomes.

**Figure 5:**
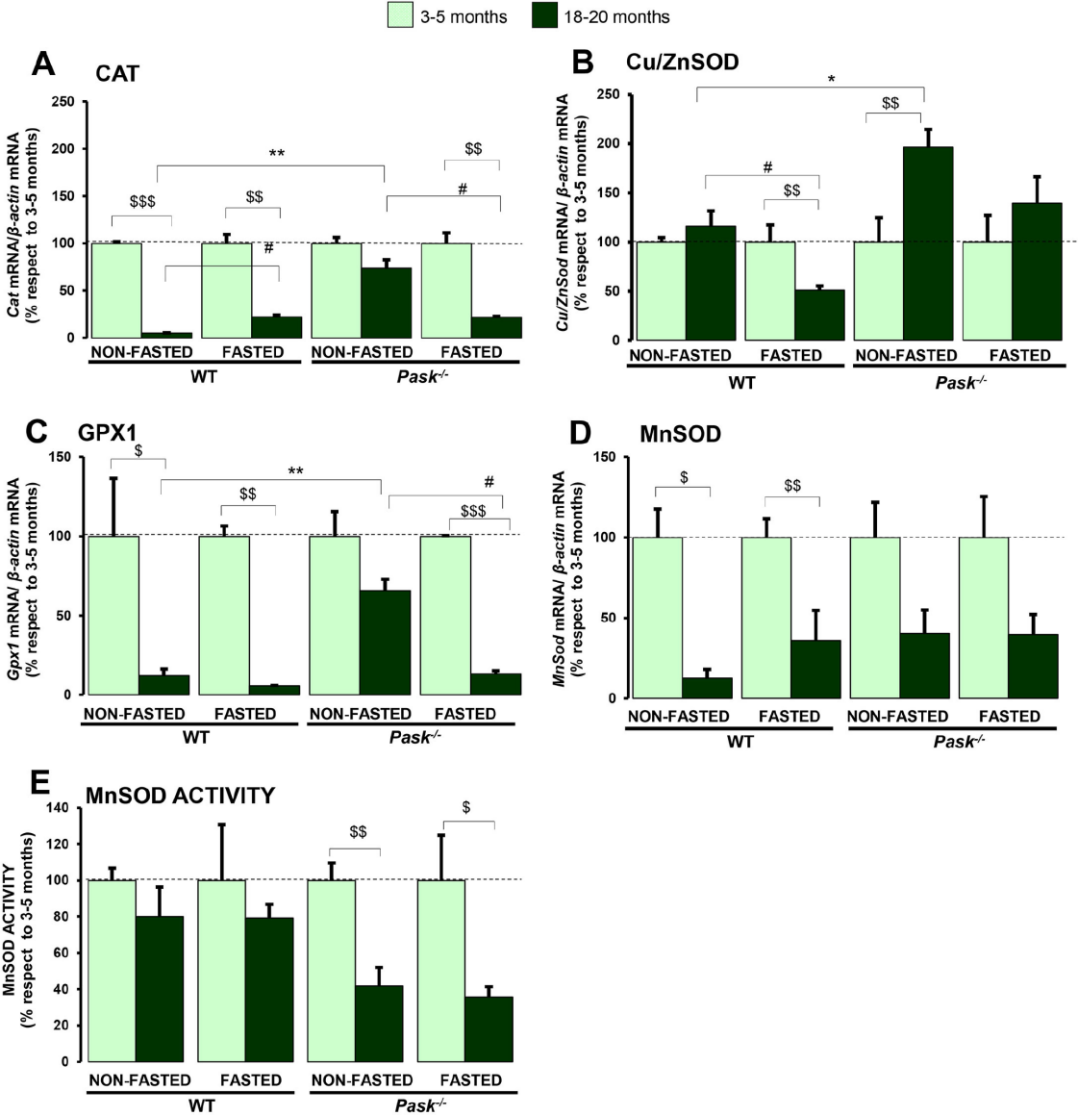
**Effects of aging and PASK deficiency on the expression and activity of antioxidant enzymes in liver.** Real-time PCR was used to analyze the expression of *Cat* (**A**)*, Cu/ZnSod* (**B**)*,* *Gpx1* (**C**) *and MnSod* (**D**) mRNA levels. The results were measured in the livers of non-fasted (NON-FASTED) and 24-h fasted (FASTED) young (3-5 months) and aged (18-20 months) wild-type (WT) and PASK-deficient (*Pask^-/-^*) mice. The mRNA levels of different genes were normalized by the mRNA of *β-actin* used as housekeeping gene. MnSOD activity in liver homogenates (**E**). Bar graphs in A-E represent the means ± SEM, the values obtained in each condition of 3-5 months was taken as 100; n = 4-5 animals per condition. ^$^ *P* < 0.05, ^$$^ *P* < 0.01, ^$$$^ *P* < 0.001 3-5 months *vs*. 18-20 months; * *P* < 0.05, ** *P* < 0.01 WT *vs*. *Pask^-/-^*; ^#^ *P* < 0.05, non-fasted *vs*. fasted.

The expression of *Cat, Gpx1* and *MnSod* was inhibited during aging under both basal and fasted conditions in WT and PASK-deficient mice. Nevertheless, PASK deficiency maintained higher levels of *Cat* and *Gpx1,* doubling the expression of *Cu/ZnSod* under basal conditions (Figure 5A–5D).

MnSOD activity in liver homogenates was similar under both basal and fasted conditions in aged mice compared to young WT. However, aged PASK-deficient mice recorded a reduced activity under both basal and fasted conditions compared to young ones. Nevertheless, the absolute values were equal in fasted aged WT and PASK-deficient mice (data not shown); this effect was due to the higher activity found in young PASK-deficient mice (Figure 5E).

### PASK deficiency increases the protein levels of NRF2, HO1 and GCLm during the aging process in response to fasting

**Figure 6:**
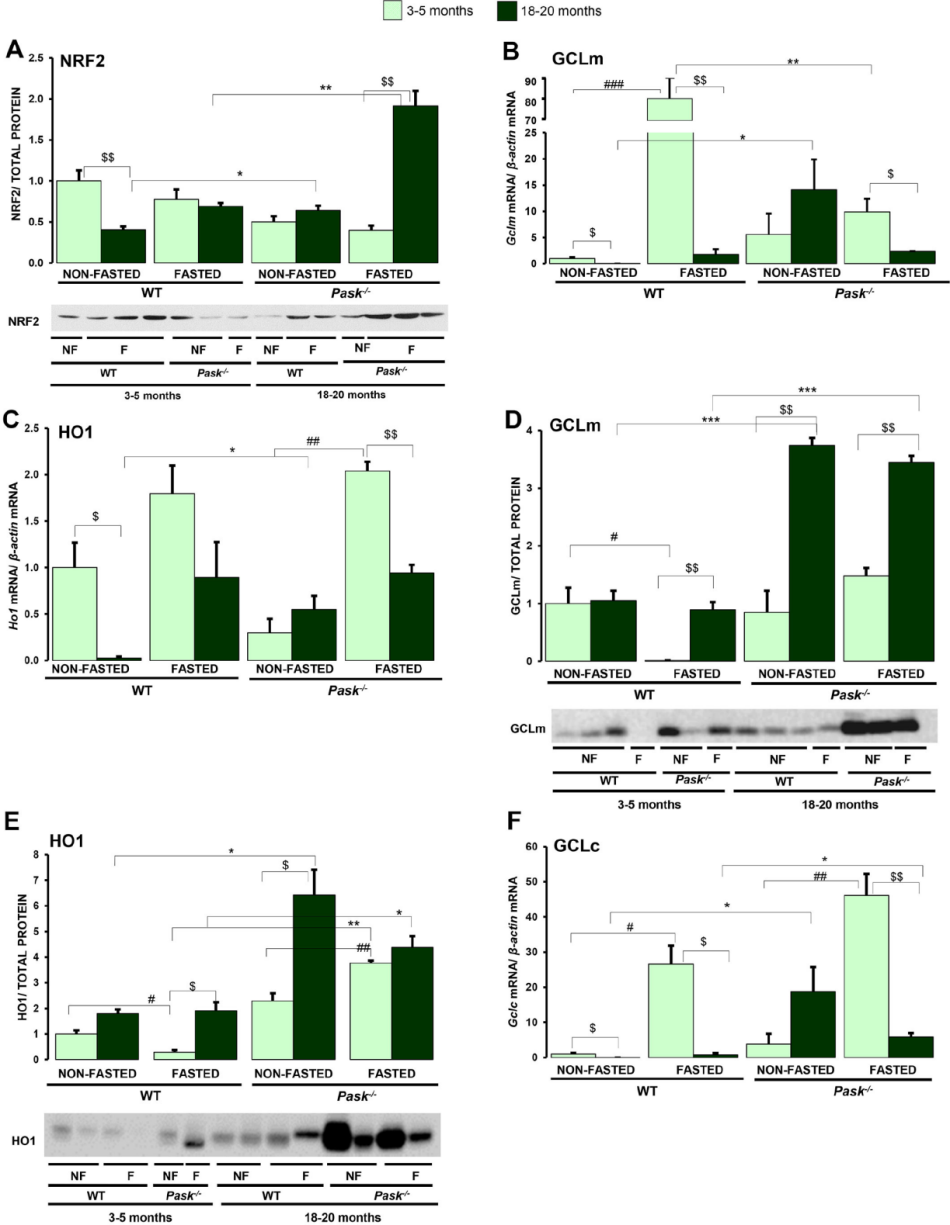
**Effects of aging and PASK deficiency on NRF2, GCLm and HO1 protein expression.** Immunoblot analysis of NRF2 (**A**), GCLm (**D**) and HO1 (**E**) in livers from young (3-5 months) and aged (18-20 months) wild-type (WT) and PASK-deficient (*Pask^-/-^*) mice. Liver lysates from non-fasted (NF) and 24-h fasted (F) mice were processed for western blot analysis. Real-time PCR was used to analyze the expression of *Gclm* (**B**), *Ho1* (**C**) and *Gclc* (**F**) mRNAs. The value obtained in 3-5-month-old non-fasted WT mice was taken as 1. Bar graphs in (**A**, **D**, **E**) represent the means ± SEM of the densitometric values normalized by total protein detected by Stain-Free (TOTAL PROTEIN) (Supplementary Figure 2); (**B**, **C**, **F**) represent the means ± SEM, and the levels of expression were normalized by the mRNA of *β-actin*, n = 4-5 animals per condition. ^$^ *P* < 0.05, ^$$^ *P* < 0.01 3-5 months *vs.* 18-20 months; * *P* < 0.05, ** *P* < 0.01, *** *P* < 0.001 WT *vs*. *Pask^-/-^*; ^#^ *P* < 0.05, ^##^ *P* < 0.01 non-fasted *vs.* fasted.

The transcription factor NRF2 functions as a major regulator of the cellular redox balance. Under high levels of ROS, it is translocated to the nucleus and induces the transcription of genes containing the Antioxidant Response Elements: *Gclc/m* (Glutamate-cysteine ligase catalytic/modifier subunits) and *Ho1* (Heme oxygenase 1). We have therefore verified whether NRF2, GCLc/m and HO1 protein expression was altered in the liver by aging in WT and PASK-deficient mice. We have shown that the gene expression of *Nrf2* increased in fasted aged PASK-deficient mice (Figure 3A). Accordingly, under fasted conditions, the NRF2 protein was also overexpressed in aged PASK-deficient mice (Figure 6A). However, aging decreased NRF2 expression under basal conditions in WT mice (Figure 6A). Similarly, either increased or similar levels of *Gclc,* *Gclm* and *Ho1* were found under basal conditions in aged PASK-deficient mice, while significant decreases in these genes were detected in aged WT under both basal and fasted conditions (Figure 6B, 6C, 6F). Furthermore, decreased expressions of *Gclm,* *Ho1* and *Gclc* were observed under fasted conditions in aged PASK-deficient mice, although this situation was accompanied by a higher protein level. GCLm and HO1 proteins levels were upregulated in aged PASK-deficient mice compared to aged WT under both fasting and non-fasting conditions (Figure 6D, 6E).

### Aging and PASK deficiency alter mitochondria remodeling and mitophagy processes

**Figure 7:**
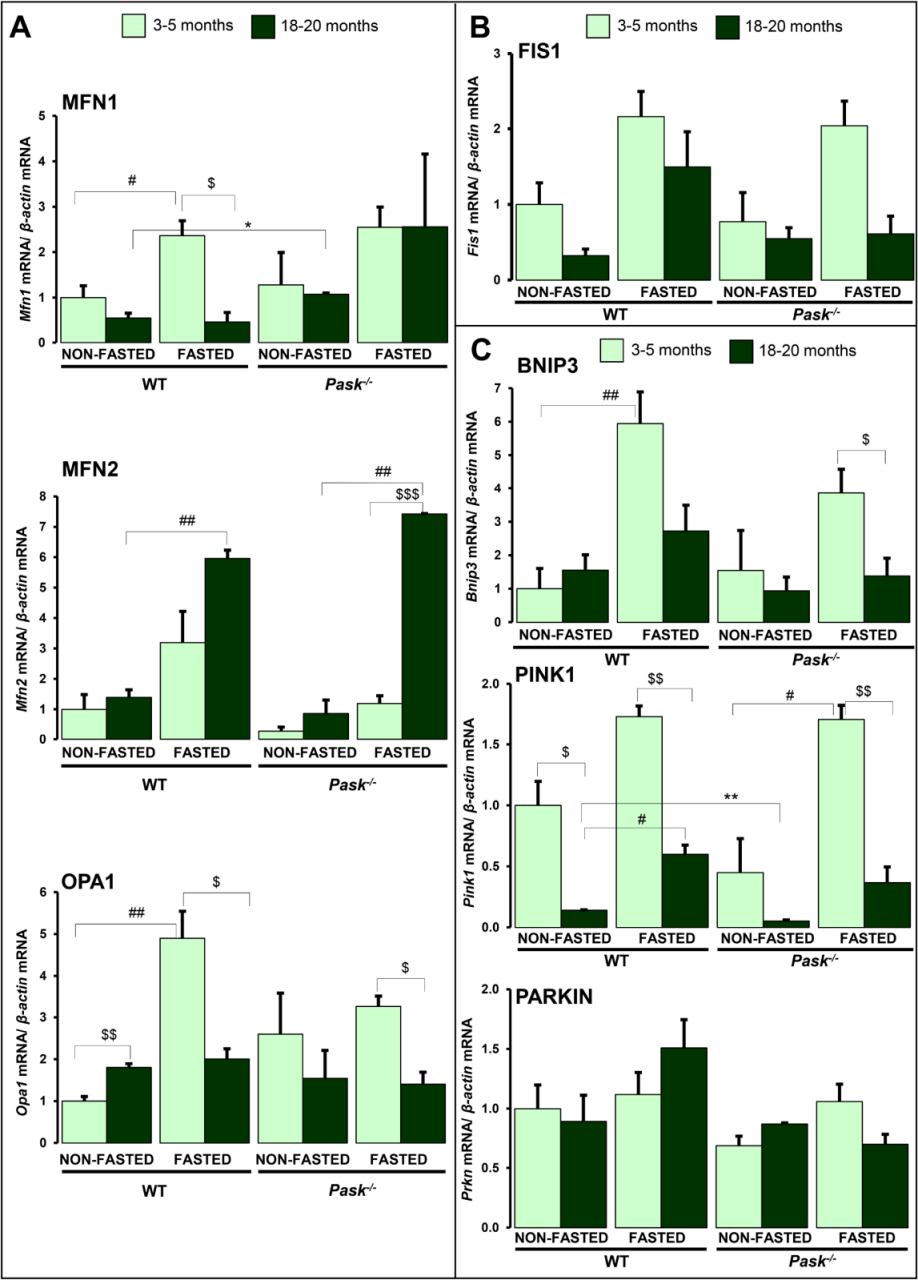
**Effects of aging and PASK deficiency on the expression of several hepatic proteins involved in mitochondria remodeling and mitophagy.** Real-time PCR was used to analyze the expression of fusion proteins *Mfn1*, *Mfn2* and *Opa1* (**A**), fission protein *Fis1* (**B**) and mitophagy proteins *Bnip3*, *Pink1* and *Parkin* (**C**) mRNA levels. The results were measured under non-fasted (NON-FASTED) and 24-h fasted (FASTED) conditions in livers from young (3-5 months) and aged (18-20 months) wild-type (WT) and PASK-deficient (*Pask^-/-^*) mice. The mRNA levels of different genes were normalized by the mRNA of *β-actin* used as housekeeping gene. The value obtained in 3-5-month-old non-fasted WT mice was taken as 1. Bar graphs represent the means ± SEM; n = 4-5 animals per condition. ^$^ *P* < 0.05, ^$$^ *P* < 0.01, ^$$$^ *P* < 0.001 3-5 months *vs.* 18-20 months; * *P* < 0.05, ** *P* < 0.01 WT *vs. Pask^-/-^*; ^*#*^ *P* < 0.05, ^*##*^ *P* < 0.01 non-fasted *vs.* fasted.

The dysfunction of mitochondrial dynamics was associated with aging. We have previously reported that PASK deficiency alters fusion and fission phenomena, playing a key role in the maintenance of viable mitochondria. Basically, the maintenance of the quality of the mitochondrial function is based on the production of new mitochondria (mitochondrial biogenesis) and the elimination of non-functional mitochondria (mitophagy). We analyzed some of the components that regulate fusion: outer mitochondrial membrane (OMM) fusion GTPase Mitofusin-1 and 2 (MFN1, MFN2), and inner mitochondrial membrane (IMM) GTPase Optic atrophy 1 (OPA1) (Figure 7A). Fission: Mitochondrial receptor protein, Fission 1 (FIS1) (Figure 7B). Mitophagy: BCL2 and adenovirus E1B 19-kDa-interacting protein (BNIP3), Phosphatase and tensin homolog (PTEN)-induced kinase 1 (PINK1) and E3 ubiquitin-protein ligase parkin (PARKIN) (Figure 7C).

**Figure 8:**
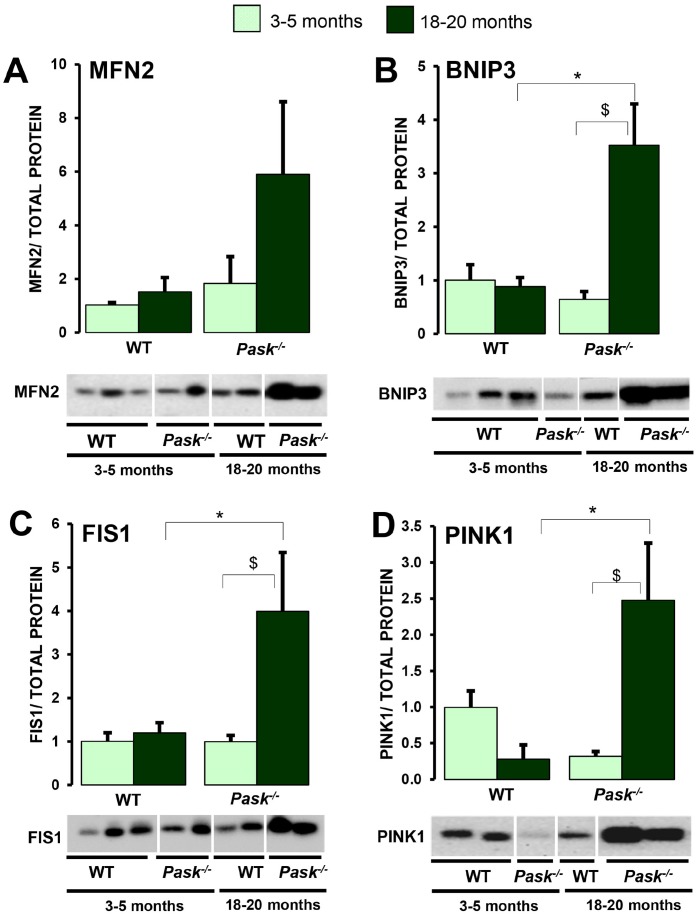
**Effects of aging and PASK deficiency on MFN2, FIS1, BNIP3 and PINK1 protein expression.** Immunoblot analysis of MFN2 (**A**), FIS1 (**B**), BNIP3 (**C**) and PINK1 (**D**) in livers from young (3-5 months) and aged (18-20 months) wild-type (WT) and PASK-deficient (*Pask^-/-^*) mice. Liver lysates from non-fasted (NF) mice were processed for western blot analysis. The value obtained in 3-5-month-old non-fasted WT mice was taken as 1. Bar graphs in (**A**–**D**) represent the means ± SEM of the densitometric values normalized by total protein detected by Stain-Free (TOTAL PROTEIN) (Supplementary Figure 2), n = 4-5 animals per condition. ^$^ *P* < 0.05 3-5 months *vs.* 18-20 months; * *P* < 0.05 WT *vs*. *Pask^-/-^*.

A decreased expression of *Pink1,* and slightly so of *Mfn1* and *Fis1,* was observed in aged WT, while only *Pink1* was lower in PASK deficiency when compared to young mice (Figure 7A–7C) under both fasted and non-fasted conditions. In addition, the stimulation of *Mnf1*, *Opa1* and *Bnip3* expression under fasted conditions disappeared in step with aging (Figure 7A, 7C) in both WT and PASK-deficient mice. However, the expression of *Pink1* and *Mfn2* increased in fasted aged WT and PASK-deficient mice, with the greater effect for *Mfn2,* whose expression was even higher than in young mice, especially in PASK-deficient specimens (Figure 7A, 7C). Nevertheless, *Prkn* expression was similar in young and aged mice in both genotypes (Figure 7C). In many cases, mRNA and protein levels did not correlate. Thus, decreased or similar expressions of *Mfn2,* *Fis1, Bnip3* and *Pink1* observed under basal conditions in aged PASK-deficient mice were accompanied by higher protein levels of MFN2, FIS1, BNIP3 and PINK1 compared to aged WT or young PASK-deficient mice (Figure 8A–8D).

### *FoxO3a,* p53 and PCNA proteins are modulated by aging and PASK dependency

**Figure 9:**
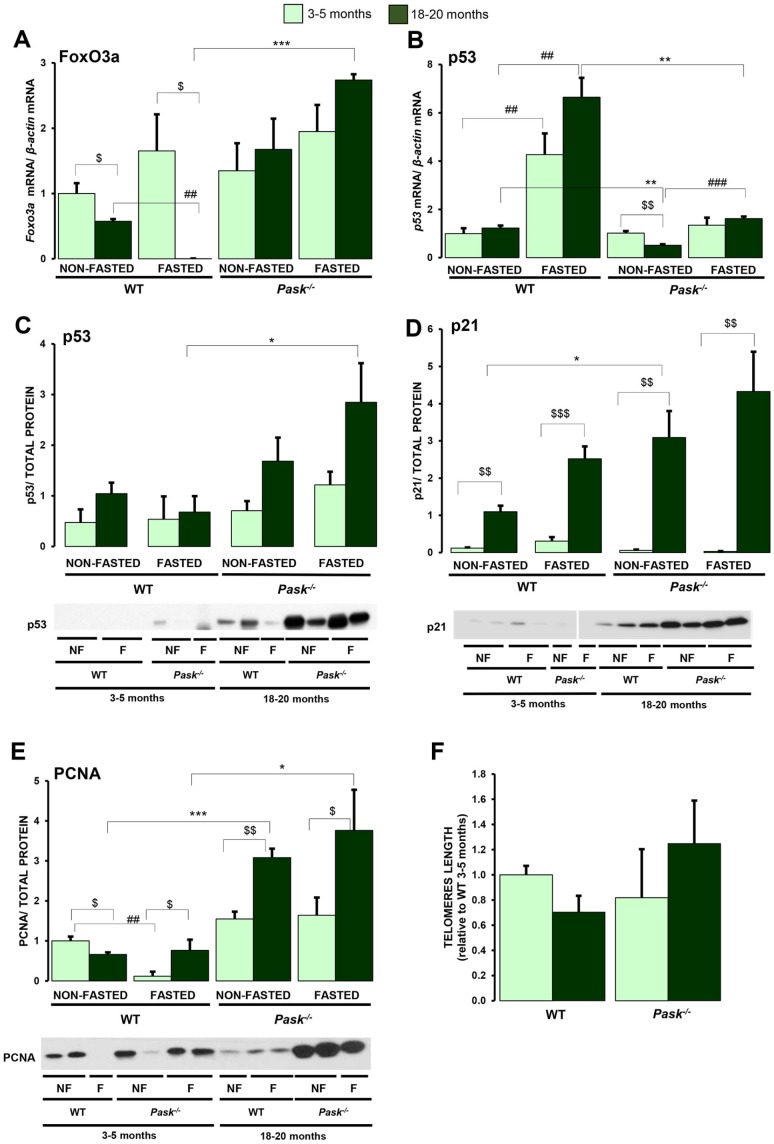
**Effects of aging and PASK deficiency on the expression of *FoxO3a* gene and p53, p21, PCNA proteins and the length of telomeres**. Real-time PCR was used to analyze the expression of *FoxO3a* (**A**) and *p53* (**B**) mRNA levels. The results were measured in the livers of non-fasted (NON-FASTED) and 24-h fasted (FASTED) young (3-5 months) and aged (18-20 months) wild-type (WT) and PASK-deficient (*Pask^-/-^*) mice. The mRNA levels of different genes were normalized by the mRNA of *β-actin* used as housekeeping gene. Immunoblot analysis of p53 (**C**), p21 (**D**), PCNA (**E**) in livers from young and aged WT and *Pask^-/-^* mice. Liver lysates from non-fasted (NF) and 24-h fasted (F) mice were processed for western blot analysis. (**C**, **D**) The value obtained in 18-20-months-old non-fasted aged WT mice was taken as 1. (**E**, **F**) The value obtained in 3-5-month-old non-fasted WT mice was taken as 1. Bar graphs represent the means ± SEM of the densitometric values normalized by total protein detected by Stain-Free (TOTAL PROTEIN) (Supplementary Figure 3) n = 4-5 animals per condition. In addition, telomere length (**F**) was measured in livers from young and aged WT and *Pask^-/-^* mice. ^$^ *P* < 0.05, ^$$^ *P* < 0.01, ^$$$^ *P* < 0.001 3-5 months *vs*. 18-20 months; * *P* < 0.05, ** *P* < 0.01 *** *P* < 0.001 WT *vs. Pask^-/-^*; ^*##*^ *P* < 0.01, ^*###*^ *P* < 0.001 non-fasted *vs.* fasted.

The aging process is also controlled by the interrelation of FoxO and p53 signaling routes involved in the mechanism of cellular survival and apoptotic responses [27, 28]. FoxO3a (Forkhead box protein O3a) is the major longevity gene identified so far, and we know that *FoxO3a* is overexpressed in PASK-deficient mice. We analyzed whether its expression was altered in liver by aging in WT and PASK-deficient mice. Our data showed that hepatic *FoxO3a* expression was reduced under basal and fasted conditions in aged WT mice. In contrast, PASK deficiency maintained *FoxO3a* gene expression slightly higher under basal conditions and upregulated it under fasted conditions compared to aged WT mice (Figure 9A), indicating that the reduction in *FoxO3a* expression by aging was PASK-dependent. However, *p53* gene expression was slightly higher in fasted aged WT compared to young mice, although p53 protein levels were similar. Nevertheless, the lower expression of *p53* gene in aged PASK-deficient mice compared to aged WT correlated significantly with higher p53 protein levels under fasted conditions (Figure 9B, 9C). In turn, p21 protein levels were almost undetectable, and no significant differences were found in either group of young mice. However, in parallel to p53, the levels of protein p21 increased markedly in aged mice in both genotypes. In particular, p21 expression tended to increase more in aged PASK-deficient mice (Figure 9D).

We have previously reported that proliferating cell nuclear antigen (PCNA) was overexpressed in PASK-deficient mice [21]. This protein is involved in DNA replication and repair. As several defects in DNA repair have been related to premature aging, we have verified PCNA levels in aged PASK-deficient mice. When the PCNA expression was analyzed, it was lower under basal conditions in aged WT and higher under fasted conditions compared to young WT, where fasting reduced it (Figure 9E). Nevertheless, the PCNA expression in aged PASK-deficient mice was always higher under all conditions compared to young PASK-deficient specimens and under all the conditions analyzed compared to WT mice (Figure 9E).

### Telomere length is maintained in aged PASK-deficient mice

The average telomere length was similar in young WT and PASK-deficient mice. Both aged WT and PASK-deficient mice maintained a similar telomere length to young mice (Figure 9F).

## DISCUSSION

Aging involves multiple changes, such as the progressive increase in blood glucose due to the insulin resistance developed by different organs. It drives the development of various metabolic disorders such as obesity and type 2 diabetes, caused by various age-related factors such as increased adiposity, decreased insulin sensitivity, and the dysfunction of pancreatic β cells [1]. A close correlation has been established between elevated oxidative stress and glucose metabolism dysfunction, as occurs in diabetic or obese patients [29, 30].

At cellular level, the aging process is controlled by molecular signaling mechanisms via FoxO, NRF2, p53, and sirtuins [28]. All these pathways share the fact they are regulated by insulin/IGF signaling, AMPK and mTOR routes that respond to nutrient levels, and they all have targets that regulate oxidative stress, metabolic homeostasis, and quality cellular housekeeping.

The PAS domains of PASK can detect intracellular oxygen, redox state, and various metabolites, and it responds by activating/inhibiting downstream routes through phosphorylation. PASK therefore controls glucose and energy metabolism homeostasis in response to metabolic requirements [31, 32]. What’s more, PASK deficiency protects against the development of obesity and the insulin resistance induced by HFD [23–25]. As these processes are affected by aging, we hypothesize that PASK plays a key role in their control.

Here, we analyze the effect of PASK deficiency in aged mice under normal and fasting conditions in order to investigate the response to conditions that promote oxidative stress [33, 34]. We describe how the liver *Pask* gene expression decreased in step with aging, being undetectable in aged WT mice. This might be an adaptive mechanism to offset certain aging impairments. In fact, this may be in agreement with the fact that throughout their lives aged PASK-deficient mice maintain the same blood glucose values as young WT mice, and do not develop insulin resistance.

The maintenance of the mitochondrial function and energy homeostasis are severely affected during the aging process, characterized by a decrease in cellular energy input [35]. Aging is thereby characterized by an increase in oxidative stress [36], changes in the levels of expression of antioxidant enzymes, and mitochondrial dysfunction, seriously affecting cellular redox homeostasis [37, 38]. The mitochondria are the main source of ROS in aging [39]. We can therefore confirm that the expression of some of the transcription factors and nuclear receptors involved in mitochondrial biogenesis (*Ppargc1a, Sirt1* and *Nrf2)* decreased in fasted aged WT mice. However, *Nrf2, Ppargc1a, Pparg* and *Sirt1* respond to 24-h fasting in aged PASK-deficient mice with a higher expression of *Nrf2*, slightly so of *Pparg,* and increased C*S* activity under fasted conditions. We likewise highlight that although PGC1a and SIRT1 protein expression decreased in both groups of aged mice, NRF2 protein expression was maintained in aged PASK-deficient mice in contrast to WT mice under basal conditions, and previous data also relate decreased NRF2 expression in liver to aging [40]. In response to aging, ROS/RNS levels increased in WT mice in accordance with the decreased expression of antioxidant enzymes. Nevertheless, PASK deficiency maintained lower levels of ROS/RNS, with a minor inhibition of the detoxification machinery.

The transcription factor NRF2 is the major regulator of the cellular redox balance [41]. The signaling system NRF2/KEAP1 is one of the main mechanisms of survival and cell defense against oxidative stress, as it regulates the transcription of redox enzymes including GCLc/m and HO1 [42, 43]. However, the efficiency of this redox system decreases with age, significantly altering the antioxidant response [40]. Accordingly, aging decreased NRF2 protein and consequently GCLm and HO1 gene expression under basal conditions in WT mice, but not in PASK-deficient mice. However, NRF2, GCLm and HO1 proteins were overexpressed, especially in aged PASK-deficient mice. The gene expression of *Gclm, Ho1* and *Nrf2* observed under fasted conditions was similar in both aged WT and PASK-deficient mice. It would be interesting to study whether PASK deficiency alters the ubiquitination and the machinery of protein degradation. Recent reports have also stated that NRF2 nuclear translocation is inhibited by non-functional PINK and by inhibitors of PI3K/Akt and p38 MAPK [44]. Akt can phosphorylate GSK3α/β and protect NRF2 from ubiquitin-proteasomal degradation [45]. Nuclear HO1 also stabilizes NRF2 from degradation [46]. Our data indicate that Akt activation was higher under fasted conditions in aged mice, as was the level of HO1 in PASK-deficient mice. The overactivation of Akt found under fasting was accompanied by higher levels of PTEN in aged mice. We have not verified whether aging and/or PASK deficiency affects the PHLPP Ser/Thr phosphatase described to specifically promote the dephosphorylation of Ser-473 Akt [47]. We cannot rule out that although PTEN levels are higher its activity could be lower under fasted conditions.

Alterations in mitochondrial dynamics during aging have been previously reported [2]. Our results confirm that mitochondrial dynamics were altered by aging, as the expression of key genes responding to nutritional availability was reduced with aging. Thus, both aged WT and PASK-deficient mice lacked the upregulated response of the expression of genes coding to fusion and fission mitochondrial proteins, and mitophagy markers (*Mnf1* and *Opa1,* *Fis1* and *Bnip3*) when nutrients are withheld. This suggests that aging blocks mitochondrial remodeling processes, and no active responses were observed in adverse nutritional deprivation situations, except in the case of *Mfn2,* in which age upregulates its expression. Interestingly, PASK deficiency promotes the protein expression of MNF2, FIS1, BNIP3 and PINK under basal conditions in aged mice. The increased expression of *Mnf2* has previously been related to high glucose oxidation rates [48]. Conversely, MFN2 liver ablation in mice was associated with high glucose production and insulin resistance and reduced autophagy [49, 50]. Reduced mitophagy has also been related, therefore, to aging-related diseases [50, 51]. PINK protects cells by inducing the elimination of damaged mitochondria through the activation of PARKIN-dependent mitophagy [52]. PASK deficiency might help to protect cells by inducing the mitophagy of damaged mitochondria, although further studies are necessary to confirm these preliminary data.

Another significant result was observed in aged PASK-deficient mice in relation to the expression of *FoxO3A.* The FoxO3a transcription factor has been considered a key mediator in the pathways involved in longevity, as it controls the expression of various antioxidant enzymes [53], preventing the development of various pathologies such as diabetes, cancer, and accelerated aging [54, 55]. Our data indicate that aging downregulates the *FoxO3a* hepatic expression in WT mice, even being undetectable under fasting conditions, in agreement with the lower gene expression of *Sirt1*. However, PASK deficiency maintained *FoxO3a* expression with aging, and its expression tends to increase under fasting conditions. FoxO3a deficiency has been shown to affect glucose metabolism [56] and cellular stress [57], while its overexpression has been related to improved hepatic insulin sensitivity in aging [53, 58]. Our results related to *FoxO3a* are therefore in accordance with the improved phenotype of aged PASK-deficient mice in terms of their insulin sensitivity and other aging-related parameters.

The Insulin/IGF pathway controls multiple cellular responses such as cell growth, proliferation and apoptosis. Additionally, this route’s key role in energy metabolism likewise influences the aging process. The activation of this pathway promotes metabolic activity, and consequently generates ROS. This effect is also induced by the inhibition of the antioxidant mechanisms, mediated by a lower expression of *FoxO3a* and *Nrf2* genes. Elevated oxidative stress thus overactivates the Akt protein, being able to stimulate cellular senescence instead of cell survival, as it could increase both the stability of the p53 protein, promoting the interruption of the cell cycle, and apoptosis [59]. Our data indicate that aged WT mice maintain the Akt protein overactivated during fasting, in contrast to young mice, sustaining the levels of p53 levels and increasing those of the p21 protein. In contrast to fasted WT, Akt activation was slightly reduced in aged PASK-deficient mice compared to young specimens, although the degree of activation was similar to fasted aged WT, and high levels of p53/p21 proteins were found. Nevertheless, in this case correlated with a higher expression of *FoxO3a* and high levels of NRF2, GCLm and HO1. It has also been reported that high levels of Akt might maintain p53 in a cytosolic location inhibiting hepatocyte senescence [60]. PASK deficiency maintains an elevated PCNA protein in aged mice, suggesting that the hepatic mechanisms involved in cell repair could be functional and active and even improved in PASK-deficient aged animals. Our data suggest that dysfunctions produced during aging in PASK-deficient mice might be connected to hormetic responses [61]. Light toxic effects might trigger beneficial compensatory responses that overcome the initial damage and improve cellular fitness. Aged PASK-deficient mice do not therefore record high blood glucose levels or insulin concentrations compared to aged WT mice. Likewise, they have increased insulin sensitivity and better glucose tolerance, which is also confirmed by a normal HOMA-IR index. In sum, PASK deficiency improves the phenotype of aged mice in many ways, such as better insulin sensitivity and glucose tolerance, as confirmed by a normal HOMA-IR index. The improvement in these parameters in aged PASK-deficient mice is accompanied by the maintenance of the longevity gene *FoxO3a* and a higher expression of NRF2, GCLm, HO1, and CS activity under fasted conditions. Moreover, some of the age-related alterations in liver feeding/fasting responses were restored in PASK-deficient mice. Under basal conditions, PASK deficiency prevented the drastically age-related decrease in the expression of antioxidant enzymes, as is the case with *Cat, Gpx1*. PASK could therefore be a good target for preventing the accumulation of cell damage in step with aging.

## MATERIALS AND METHODS

### Experimental animals and treatments

All the procedures involving animals were approved by the appropriate Institutional Review Committee, reference 315/15, and met the guidelines for the care of animals specified by the European Community. The animals used were C57BL/6J wild-type (WT) and PASK-deficient (*Pask^-/-^*) mice crossed into C57BL/6J for at least 12 generations [62], and they were male (25-30 g), at the ages of 3-5 months (young), 12 months, and 18-20 months. The animals were fed *ad libitum* with a standard pellet diet and housed at a constant temperature (21º C) on a 12-hour light-dark cycle, with lights on at 8 am.

Both groups of mice, *Pask^-/-^* and WT, were kept under standard feeding conditions (*ad libitum*) (non-fasted) or 24-h fasted. The mice were then decapitated, and their liver was immediately frozen.

### Real-time polymerase chain reaction

Liver total RNA from WT and *Pask^-/-^* mice was extracted with TRIzol (Life Technologies, Barcelona, Spain). RNA integrity was tested with the Nanodrop 2000 (Thermo Scientific), and cDNA synthesis was developed using the High-capacity cDNA archive kit (Applied Biosystems), using 2 μg of RNA as template, following the manufacturer’s instructions. Four microliters of a 1:10 dilution of the cDNA was used as a template for the polymerase chain reaction (PCR). Either TaqMan® Assay (Applied Biosystems, Foster City, CA) or SYBR Green® Assay (Applied Biosystems) was used to quantify the mRNA levels by real-time PCR in a 7300HT Fast Real-Time PCR System (Applied Biosystems). The details of the primers and probes are listed in Supplementary Table 1. The PCR conditions were 50º C for 2 min, 95º C for 10 min, followed by 40 cycles at 95º C for 15 s, and 60º C for 1min. *18S* and *β-actin* housekeeping genes were used for normalization. In the case of SYBR Green Assay, a standard curve was first generated in each real-time PCR assay by tenfold serial dilutions of the cDNA samples.

For mtDNA quantification, liver total DNA from WT and *Pask^-/-^* mice was extracted in a buffer containing 10% Chelex 100 resin (Bio-Rad) and digested with Proteinase K (10 mg/mL) for one hour at 55ºC. RT-PCR was performed from total DNA using primers to detect mitochondrial DNA coding for *16S* rRNA, and nuclear DNA coding for *β-actin* (Supplementary Table 1).

### Telomere length analysis

Telomer length was assayed through the use of, ScienCell's Relative Mouse Telomere Length Quantification qPCR Assay Kit (RMTLQ) according to the manufacturer’s instructions. Total liver DNA from WT and *Pask^-/-^* mice was extracted as described in this manuscript. *β-actin* amplification served as a reference for data normalization.

### Liver protein detection by western blot

The analysis of protein expression by western blotting involved a tiny piece of frozen liver (~150 mg) being immediately lysed in a RIPA buffer (PBS, 1 % NP-40, 0.5% sodium deoxycholate, 1mM PMSF, 10 mM Leupeptin, 1mM NA_2_VO_4_, 25mM Na_4_P_2_O_7_, 10mM FNa) and protease inhibitor cocktail (Roche Diagnostics, Mannheim, Germany). The tissues were immediately exposed to microwave irradiation for 5s, and then homogenized [63]. After their transfer to a PVDF membrane (Immun-Blot@ PVDF, Bio-Rad), total and activated forms of proteins were detected by western blotting using the antibodies described (Supplementary Table 2), followed by incubation with the specific secondary antibodies bound to HRP. Finally, the blots were quantified using Quantity One software (Bio-Rad, GS-800 Densitometer). Stain-Free staining was used as a loading control [64, 65] (Supplementary Figure) as an alternative to Ponceau staining, for example. The detection of proteins on Stain-Free gels is based on the modification of tryptophan residues from proteins using a trihalo compound. After their transfer to a membrane, the modified tryptophans give off a fluorescent signal when illuminated with UV light that is proportional to protein load, which can be readily detected and quantified for use as loading control.

### Measurement of total reactive species levels

Total reactive species levels (ROS/RNS) in liver were measured using the OxiSelect in vitro ROS/RNS Assay Kit (Green Fluorescence) (Cell Biolabs, INC.), following the manufacturer’s instructions. The ROS/RNS values were extrapolated from a standard curve, and involved tenfold serial dilutions of 1 mM DCF (2′,7′-diclorodihidrofluorescein).

### Determination of citrate synthase (CS) and manganese superoxide dismutase (MnSOD) activities

The citrate synthase (CS) activity of liver samples under the conditions indicated was measured using the Citrate Synthase Activity Assay Kit from Sigma (MAK193), following the manufacturer’s instructions. CS activity was determined using a coupled enzyme reaction, resulting in a colorimetric (412 nm) product proportional to the enzymatic activity. The reaction was measured every 5 min for 40 min at 25ºC, considering the penultimate reading to be the final result. CS activity was stated as a percentage. To measure liver cytosolic SOD activity, a commercially available kit (STA-340, Cell Biolabs, INC.) was used according to the manufacturer’s protocol. Absorbance was measured at 490 nm after one-hour incubation at 37°C. SOD activity was stated as a function of inhibition percentage.

### Serum analysis

Blood plasma insulin levels were measured using a competitive ELISA Kit (Millipore, MA, USA), following the manufacturer’s instructions. Plasma triglyceride (TG) and total cholesterol (TCH) were measured using a Roche cobas b 101 Lipid Control instrument (Roche Diagnostics, Basel, Switzerland), following the manufacturer´s instructions.

### Glucose and insulin tolerance tests (GTT/ITT)

For GTT, experimental animals were fasted overnight, and an intraperitoneal (IP) injection was used to administer glucose (2 g/kg body weight). At the indicated times, tail vein blood was sampled for measuring glucose with a Glucometer Elite meter (Bayer Corp. Elkhart, IN, USA). For ITT, experimental animals were fasted for four hours, and an IP injection was used to administer insulin (1 U/kg body weight), with blood glucose levels being measured at the specified times.

### HOMA-IR

To evaluate insulin resistance, HOMA-IR was calculated from the fasting concentrations of insulin and glucose using the following formula: fasting serum insulin (mU/L) × fasting plasma glucose (mmol/L)/22.5. As the values were higher than 1, this meant a greater susceptibility to developing insulin resistance.

### Statistical analyses

Data were presented as the means ± SEM. For experiments with multiple comparisons, differences between groups were first tested with three- or two-way ANOVA, followed by pairwise t-test comparisons with Tukey’s post hoc test to determine the differences among groups. Data were considered statistically significant at P ≤ 0.05. Statistical analyses were performed using GraphPad Prism software.

## Supplementary Material

Supplementary Figures

Supplementary Tables
