# Supplementary material for: PAS kinase deficiency reduces aging effects in mice
Source: Aging (Albany NY). 2020 Jan 23;12(3):2275–301. doi: 10.18632/aging.102745 (PMC7041766; doi:10.18632/aging.102745)
Supplement: Supplementary Figures [file aging-12-102745-s002..pdf]

SUPPLEMENTARY FIGURES

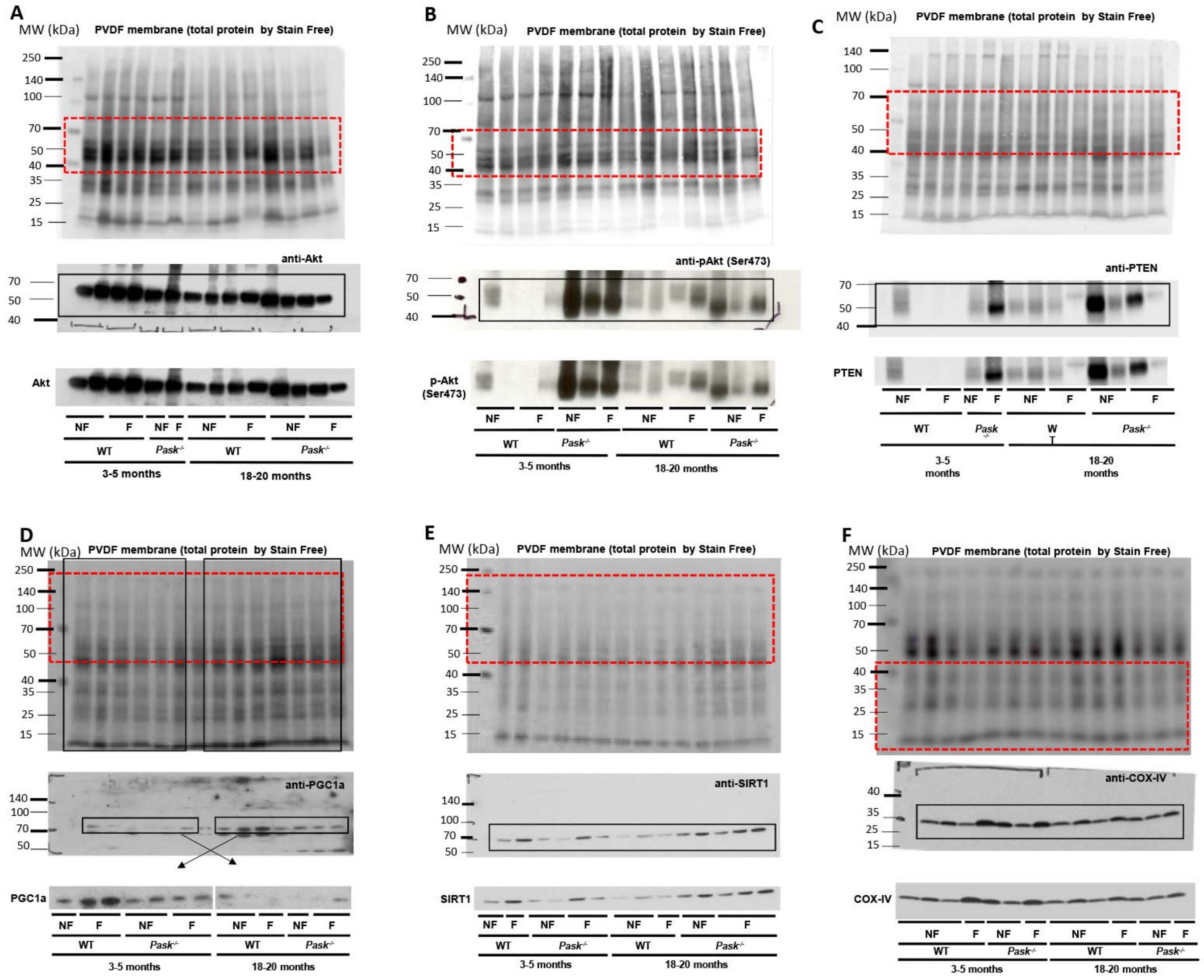

Supplementary Figure 1. Full-length stain free PVDF membranes and blot images of all the figures presented in the text. The membranes were fragmented before antibody incubation and incubate with each different antibodies (dotted red line). The membranes were cropped, in order to save the use of large amounts of expensive antibodies. The regions of membrane incubated, were according to the molecular weight of protein to be detected. The black line boxes indicate the fragment of the membrane with the protein of interest. The boxed bands correspond to the cropped blots that appear in the final main figure. The other bands discarded were controls or treatments without interest for this work. Final cropped blots are displayed. NF: NON-FASTED; F: FASTED.

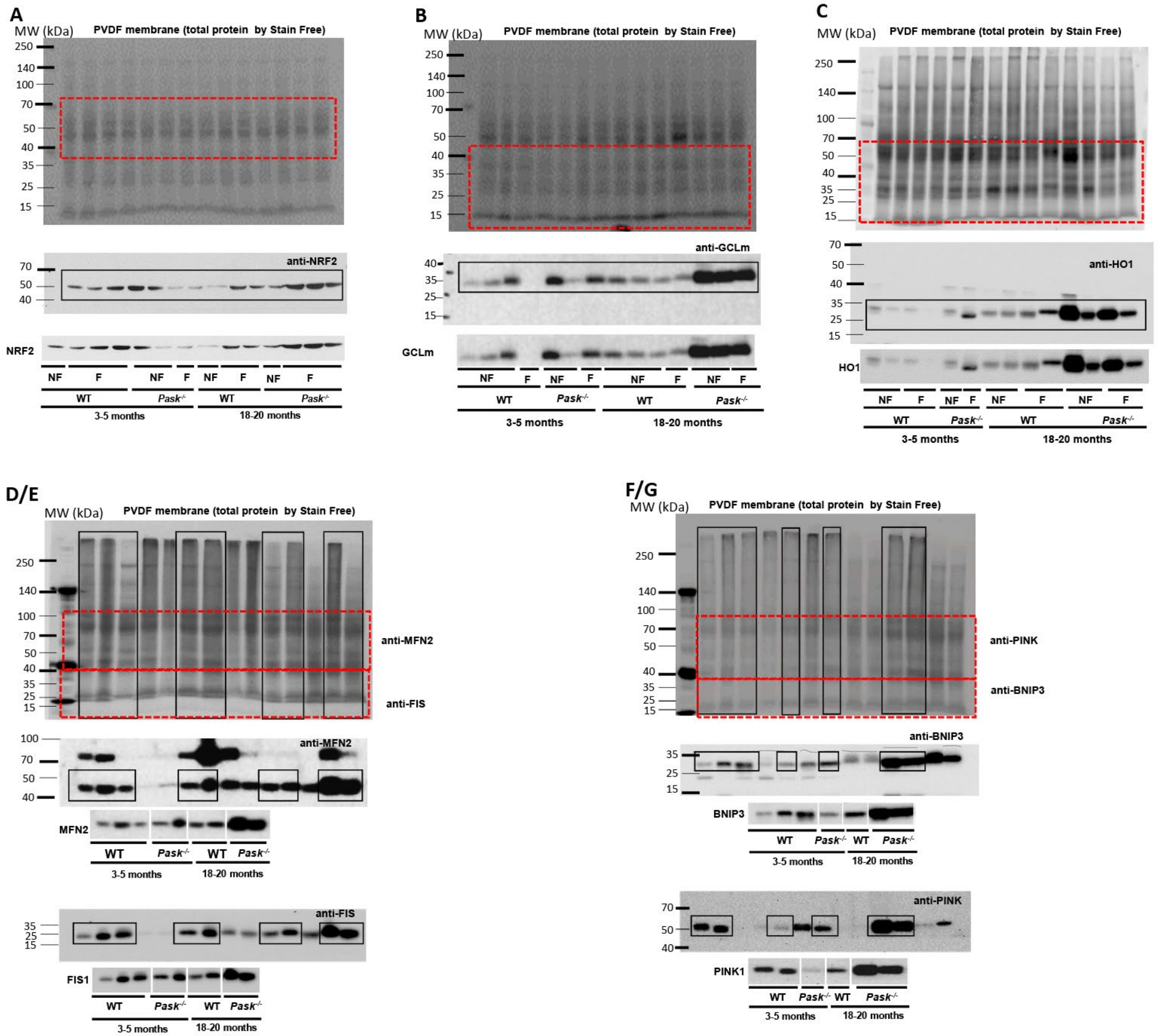

Supplementary Figure 2. Full-length stain free PVDF membranes and blot images of all the figures presented in the text. The membranes were fragmented before antibody incubation and incubate with each different antibodies (dotted red line). The membranes were cropped, in order to save the use of large amounts of expensive antibodies. The regions of membrane incubated, were according to the molecular weight of protein to be detected. The black line boxed indicate the fragment of the membrane with the protein of interest. The boxes bands correspond to the cropped blots that appear in the final figure. The other bands discarded were controls or treatments without interest for this work. Final cropped blots are displayed. NF: NON-FASTED; F: FASTED.

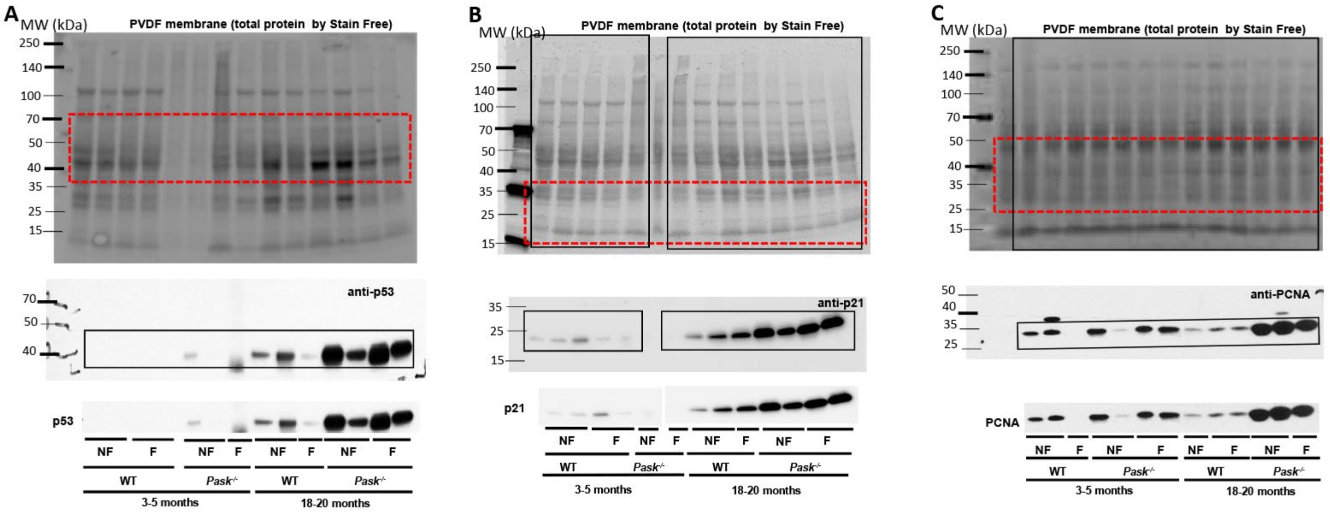

Supplementary Figure 3. Full-length stain free PVDF membranes and blot images of all the figures presented in the text. The membranes were fragmented before antibody incubation and incubate with each different antibodies (dotted red line). The membranes were cropped, in order to save the use of large amounts of expensive antibodies. The regions of membrane incubated, were according to the molecular weight of protein to be detected. The black line boxed indicate the fragment of the membrane with the protein of interest. The boxes bands correspond to the cropped blots that appear in the main figure. The other bands discarded were controls or treatments without interest for this work. (*) This fragment of membrane indicates that it was turned. Final cropped blots are displayed. NF: NON-FASTED; F: FASTED.
