# Supplementary material for: PAS kinase deficiency reduces aging effects in mice
Source: Aging (Albany NY). 2020 Jan 23;12(3):2275–301. doi: 10.18632/aging.102745 (PMC7041766; doi:10.18632/aging.102745)
Supplement: Supplementary Tables [file aging-12-102745-s001..pdf]

SUPPLEMENTARY TABLES

Supplementary Table 1. Identification of primers used in the different gene expression assays.

Real-time polymerase chain reaction (TAQMAN® ASSAY)

Gene (protein)	Probe identification (Taqman® Assay)
18S (18S)	Hs99999901_s1
Pask (PASK)	Mm00435916_m1

Real-time polymerase chain reaction (SYBR GREEN® ASSAY)

Gene (protein)	Mouse forward primer	Mouse reverse primer
16S rRNA (16S)	5′-CCGCAAGGGAAAGATGAAAGAC-3′	5′-TCGTTTGGTTTCGGGGTTTC-3′
Actb (β-ACTIN)	5′-CTCTCTTCCAGCCTTCCTTC-3′	5′-GGTCTTTACGGATGTCAACG-3′
Bnip3 (BNIP3)	5′-CAGCATGAATCTGGACGAAG-3′	5′-ATCTTCCTCAGACAGAGTGC-3′
Cat (CAT)	5′-GAATGGCTATGGTCCACACA-3′	5′-CAAGTTTTTGATGCCCTGGT-3′
CoxIV (COXIV)	5′-TGAATGGAAGACAGTTGTGGG-3′	5′-GATCGAAAGTATGAGGGATGGG-3′
Cs (CS)	5′-GGGACTTGTGTATGAGACTTCG-3′	5′-AGCCAAAATAAGCCCTCAGG-3′
Cu/ZnSod (Cu/ZnSOD)	5′-GGTGGTCCACGAGAAACAAG-3′	5′-CAATCACACCACAAGCCAAG-3′
Fis1 (FIS1)	5′-GCCCCTGCTACTGGACCAT-3′	5′-CCCTGAAAGCCTCACACTAAGG-3′
FoxO3a (FoxO3a)	5′-TACGAGTGGATGGTGCGCTG-3′	5′-AGGTTGTGCCGGATGGAGTTC-3′
Gclc (GCLc)	5′- AGGCTCTCTGCACCATCACT-3′	5′- CTCTGGGTTGGGTCTGTGTT-3′
Gclm (GCLm)	5′-TGTGTGATGCCACCAGATTT-3′	5′- GATGATTCCCCTGCTCTTCA-3′
Gpx (GPx)	5′-TGCAATCAGTTCGGACATC-3′	5′-CACCTCGCACTTCTCAAACA-3′
Ho1 (HO1)	5′-AGCCCCACCAAGTTCAAACA-3′	5′-CATCACCTGCAGCTCCTCCA-3′
Mcad (MCAD)	5´-TTCGAAGACGTCAGAGTGC-3´	5´-GCGACTGTAGGTCTGGTTC-3´
Mfn1 (MFN1)	5′-TCTCCAAGCCCAACATCTTCA-3′	5′-ACTCCGGCTCCGAAGCA-3′
Mfn2 (MFN2)	5′-AGGAGTGGTGTGGAAGGCAG-3′	5′-ACAAACTGGCGCTTGAAGG-3′
MnSod (MnSOD)	5′-AAGGAGCAAGGTCGCTTACA-3′	5′-ACACATCAATCCCCAGCAGT-3′
Nrf2 (NRF2)	5′-CTACTCCCAGGTTGCCCACA-3′	5′-CGACTCATGGTCATCTACAAATGG-3′
Opa1 (OPA1)	5′-CTGGAAGAATCGGACCCAAG-3′	5′-AGGTTCTTCCGGACTGTGGT-3′
p53 (p53)	5′-CCGTGTTGGTTCATCCCTGTA-3′	5′-TTTTGGATTTTTAAGACAGAGTC-3′
Pink1 (PINK1)	5′-CTACCGCTTCTTCCGCCAGT -3′	5′-AGCCCGAAGGCCAGAAAGAC-3′
Pparg (PPARg)	5′-GTGCCAGTTTGCATCCGTAGA-3′	5′-GGCCAGCATCGTGTAGATGA-3′
Ppargc1a (PGC1a)	5′-ATGTGTCGCCTTCTTGCTCT-3′	5′-ATCTACTGCCTGGGGACCTT-3′
Sirt1 (SIRT1)	5′-TTGTGAAGCTGTTCGTGGAG-3′	5′-GGCGTGGAGGTTTTTCAGTA-3′

Supplementary Table 2. Antibodies and conditions used for western blot assays.

Antibody	Host	Manufacturer	Dilution used
Anti-Akt	Mouse	Cell Signaling Technology, Danvers, MA, USA	1: 1000
Anti-BNIP3	Mouse	Santa Cruz Biotechnology, California, USA	1: 1000
Anti-COXIV	Mouse	GeneTex, Irvine, USA	1: 2000
Anti-FIS	Mouse	Santa Cruz Biotechnology, California, USA	1: 1000
Anti-GCLm	Rabbit	Sigma Aldrich, Madrid, Spain	1: 5000
Anti-HO1	Mouse	Mouse Santa Cruz Biotechnology, California, USA	1: 1000
Anti-MFN2	Mouse	Santa Cruz Biotechnology, California, USA	1: 1000
Anti-Mouse-HRP	Goat	Bethyl Laboratories, Montgomery, USA	1: 5000
Anti-NRF2	Rabbit	GeneTex, Irvine, EEUU	1: 1000
Anti-p53	Rabbit	Cell Signaling Technology, Danvers, MA, USA	1: 1000
Anti-PCNA	Mouse	Abcam, Cambrigde, United Kingdom	1: 1000
Anti-PGC1a	Rabbit	Milipore Iberica, Madrid, Spain	1: 1000
Anti-phospho-Akt1/PKBα (Ser473)	Rabbit	Millipore Iberica, Madrid, Spain	1: 1000
Anti-PINK1	Mouse	Santa Cruz Biotechnology, California, USA	1: 1000
Anti-PTEN	Rabbit	Milipore Iberica, Madrid, Spain	1: 500
Anti-Rabbit-HRP	Goat	Milipore Iberica, Madrid, Spain	1: 5000
Anti-SIRT1	Rabbit	Santa Cruz Biotechnology, California, USA	1: 500
